# Supplementary material for: A Likelihood Approach for Real-Time Calibration of Stochastic Compartmental Epidemic Models
Source: PLoS Comput Biol. 2017 Jan 17;13(1):e1005257. doi: 10.1371/journal.pcbi.1005257 (PMC5240920; doi:10.1371/journal.pcbi.1005257)
Supplement: S1 File — (TAR.GZ) [file pcbi.1005257.s014.tar.gz › HSPH_Online-SI-Revision/MSS10/n100-mild/n100-mild_NEW2_table0.pdf]

1	newly 3 weeks spec	2866.	2503.26	{2201., 2845.}	12.8248	0.0614152	newly 3 weeks cumul	9776.	9116.77	{8132., 10099.}	8.16438	0.0377648	newly 5 weeks spec	1628.	1384.01	{1164., 1574.}	15.422	0.075098	newly 5 weeks cumul	13712.	12420.8	{11036., 13606.}	9.79449	0.0457959	newly 52 weeks cumul	16805.	15431.8	{14126., 16604.}	8.59399	0.0397148
2	newly 3 weeks spec	3141.	2323.04	{2044., 2580.}	26.0414	0.132639	newly 3 weeks cumul	11118.	9370.3	{8563., 10180.}	15.7336	0.0754483	newly 5 weeks spec	1875.	1124.01	{945., 1298.}	40.0528	0.226252	newly 5 weeks cumul	15610.	12149.	{10903., 13204.}	22.1718	0.110054	newly 51 weeks cumul	19660.	14231.9	{12867., 15496.}	27.6099	0.141786
3	newly 3 weeks spec	2615.	2504.09	{2138., 2825.}	8.98088	0.0408739	newly 3 weeks cumul	10083.	9852.73	{8633., 10881.}	6.91887	0.0309337	newly 5 weeks spec	1275.	1226.18	{989., 1436.}	11.222	0.051362	newly 5 weeks cumul	13206.	12881.	{11404., 14337.}	6.93783	0.0311382	newly 54 weeks cumul	16122.	15146.1	{13133., 16722.}	8.07096	0.0372531
4	newly 3 weeks spec	3002.	1742.78	{1439., 1960.}	41.946	0.242285	newly 3 weeks cumul	14624.	11053.5	{9594., 12262.}	24.4151	0.123504	newly 5 weeks spec	1149.	412.04	{283., 512.}	64.1393	0.469025	newly 5 weeks cumul	17569.	12323.1	{10723., 13503.}	29.859	0.156398	newly 58 weeks cumul	18847.	12705.3	{11143., 13752.}	32.5871	0.173992
5	newly 3 weeks spec	2761.	2231.63	{1993., 2476.}	19.1862	0.0940496	newly 3 weeks cumul	10134.	8856.38	{8110., 9515.}	12.6562	0.0597389	newly 5 weeks spec	1641.	1107.6	{929., 1290.}	32.5046	0.174017	newly 5 weeks cumul	14065.	11585.2	{10638., 12540.}	17.6314	0.0852022	newly 54 weeks cumul	17712.	13716.5	{12596., 14763.}	22.5582	0.112026
6	newly 3 weeks spec	2631.	2196.47	{1896., 2456.}	16.9111	0.0829799	newly 3 weeks cumul	10483.	9233.3	{8139., 10192.}	12.2097	0.0579435	newly 5 weeks spec	1399.	984.06	{756., 1168.}	30.0343	0.161396	newly 5 weeks cumul	13754.	11724.2	{10482., 13044.}	14.8574	0.0715897	newly 52 weeks cumul	16963.	13401.3	{12066., 15001.}	20.9967	0.104465
7	newly 3 weeks spec	3036.	2982.33	{2618., 3713.}	9.99308	0.0429631	newly 3 weeks cumul	13981.	13695.2	{12264., 15429.}	7.45283	0.0327633	newly 5 weeks spec	924.	1026.	{795., 1444.}	17.8528	0.0679893	newly 5 weeks cumul	16613.	16516.5	{14803., 19600.}	7.68946	0.0330254	newly 57 weeks cumul	17582.	17799.8	{16123., 21532.}	7.56035	0.0316424
8	newly 3 weeks spec	2036.	1997.16	{1721., 2217.}	7.91749	0.0351756	newly 3 weeks cumul	8038.	8146.22	{7234., 9011.}	6.90072	0.0297969	newly 5 weeks spec	1057.	944.43	{792., 1119.}	14.2583	0.0676197	newly 5 weeks cumul	10545.	10492.9	{9311., 11548.}	6.43689	0.0282289	newly 51 weeks cumul	12212.	12262.8	{10945., 13428.}	6.40272	0.0277966
9	newly 3 weeks spec	3348.	2420.8	{2126., 2657.}	27.6941	0.142313	newly 3 weeks cumul	14156.	11840.7	{10963., 12689.}	16.3558	0.0783544	newly 5 weeks spec	1368.	831.33	{706., 942.}	39.2303	0.219189	newly 5 weeks cumul	17721.	14118.6	{13223., 15083.}	20.3283	0.0994158	newly 56 weeks cumul	20118.	15183.3	{14164., 16216.}	24.5288	0.122854
10	newly 3 weeks spec	2716.	2039.52	{1728., 2351.}	24.986	0.127899	newly 3 weeks cumul	9804.	8308.61	{7250., 9355.}	15.4016	0.0744708	newly 5 weeks spec	1557.	977.97	{804., 1127.}	37.2004	0.207186	newly 5 weeks cumul	13469.	10730.9	{9382., 12057.}	20.3289	0.100677	newly 50 weeks cumul	16876.	12599.3	{11143., 13792.}	25.3421	0.128802
11	newly 3 weeks spec	3997.	2657.38	{2304., 2939.}	33.5156	0.179095	newly 3 weeks cumul	15533.	12234.7	{10886., 13585.}	21.2339	0.105306	newly 5 weeks spec	1963.	919.83	{769., 1063.}	53.1416	0.332671	newly 5 weeks cumul	20137.	14739.4	{13321., 16011.}	26.8044	0.136834	newly 54 weeks cumul	22930.	15924.4	{14520., 17261.}	30.5519	0.159351
12	newly 3 weeks spec	3071.	2417.31	{2160., 2654.}	21.2859	0.105718	newly 3 weeks cumul	10922.	9322.21	{8541., 10021.}	14.6474	0.0697766	newly 5 weeks spec	2036.	1284.95	{1065., 1459.}	36.8885	0.203397	newly 5 weeks cumul	15499.	12404.6	{11370., 13291.}	19.9653	0.0978737	newly 50 weeks cumul	21968.	15152.5	{13339., 16153.}	31.0249	0.162735
13	newly 3 weeks spec	3153.	2316.29	{2019., 2618.}	26.5369	0.136346	newly 3 weeks cumul	12554.	10372.1	{8957., 11648.}	17.5076	0.0857127	newly 5 weeks spec	1478.	864.81	{730., 1017.}	41.4878	0.2364	newly 5 weeks cumul	16279.	12689.	{11345., 13980.}	22.0529	0.110018	newly 55 weeks cumul	18456.	13897.2	{12564., 15308.}	24.7007	0.124562
14	newly 3 weeks spec	2684.	2438.4	{2253., 2610.}	9.25335	0.042886	newly 3 weeks cumul	12513.	12416.8	{11497., 13147.}	3.97946	0.0175005	newly 5 weeks spec	929.	780.61	{677., 880.}	16.1001	0.0782344	newly 5 weeks cumul	15065.	14588.8	{13607., 15358.}	4.20478	0.0188849	newly 57 weeks cumul	16544.	15527.7	{14541., 16345.}	6.21089	0.0282576
15	newly 3 weeks spec	3944.	2838.37	{2513., 3140.}	28.0332	0.145475	newly 3 weeks cumul	16141.	13296.4	{12065., 14514.}	17.6234	0.0855765	newly 5 weeks spec	1651.	993.71	{808., 1184.}	39.8116	0.22876	newly 5 weeks cumul	20293.	16000.9	{14162., 17446.}	21.1506	0.104743	newly 54 weeks cumul	22727.	17253.7	{15723., 18627.}	24.0829	0.121306
16	newly 3 weeks spec	2270.	2173.7	{1829., 2485.}	9.99471	0.0467798	newly 3 weeks cumul	7578.	7670.32	{6587., 8647.}	9.17947	0.0401638	newly 5 weeks spec	1786.	1294.64	{1002., 1532.}	27.5118	0.148635	newly 5 weeks cumul	11349.	10682.4	{9237., 11870.}	8.81346	0.0417558	newly 50 weeks cumul	16325.	13924.1	{12211., 15635.}	14.707	0.0727929
17	newly 3 weeks spec	4099.	2287.53	{2029., 2532.}	44.193	0.25474	newly 3 weeks cumul	15754.	11517.6	{10228., 12747.}	26.8912	0.137331	newly 5 weeks spec	1851.	730.55	{611., 848.}	60.5321	0.407515	newly 5 weeks cumul	20371.	13570.9	{12348., 14865.}	33.3815	0.177458	newly 56 weeks cumul	22951.	14443.	{13311., 15662.}	37.0703	0.202075
18	newly 3 weeks spec	3126.	2627.01	{2021., 3109.}	16.9331	0.0860712	newly 3 weeks cumul	17638.	15563.9	{13223., 17411.}	12.2476	0.0592126	newly 5 weeks spec	665.	568.26	{357., 766.}	22.7308	0.11934	newly 5 weeks cumul	19872.	17400.7	{14559., 19578.}	12.8661	0.0625914	newly 60 weeks cumul	20225.	17858.4	{14715., 20120.}	12.2555	0.0595901
19	newly 3 weeks spec	3015.	2652.07	{2401., 2854.}	12.3718	0.0583856	newly 3 weeks cumul	11397.	10293.4	{9270., 11059.}	10.0615	0.0468362	newly 5 weeks spec	1523.	1282.74	{1070., 1484.}	16.4202	0.0808928	newly 5 weeks cumul	15147.	13471.1	{12367., 14554.}	11.2227	0.0525663	newly 51 weeks cumul	18357.	15866.	{14187., 17179.}	13.6124	0.0645618
20	newly 3 weeks spec	3891.	3725.12	{3044., 4390.}	13.3344	0.0588181	newly 3 weeks cumul	17885.	18282.5	{15584., 21014.}	9.48588	0.0405141	newly 5 weeks spec	1246.	949.55	{606., 1329.}	32.1292	0.171633	newly 5 weeks cumul	21430.	21168.3	{18224., 24064.}	9.25973	0.0402556	newly 60 weeks cumul	22520.	22018.7	{19177., 25360.}	9.79161	0.0426248
21	newly 3 weeks spec	3638.	3611.02	{2055., 4254.}	18.0225	0.0871982	newly 3 weeks cumul	16322.	16841.1	{12877., 18887.}	12.4686	0.0551895	newly 5 weeks spec	1041.	1160.53	{437., 1509.}	37.7032	0.174179	newly 5 weeks cumul	19258.	20098.9	{14309., 22648.}	15.7359	0.0695614	newly 60 weeks cumul	20284.	21463.5	{14625., 24389.}	18.0858	0.079313
22	newly 3 weeks spec	3362.	1889.21	{1602., 2117.}	43.807	0.252614	newly 3 weeks cumul	10760.	7839.37	{6826., 8602.}	27.1434	0.1392	newly 5 weeks spec	2166.	896.32	{744., 1053.}	58.6187	0.386855	newly 5 weeks cumul	15662.	10063.8	{8922., 11094.}	35.7436	0.193602	newly 50 weeks cumul	22682.	11758.9	{10621., 12888.}	48.1575	0.286643
23	newly 3 weeks spec	3951.	5588.33	{5365., 5838.}	41.4409	0.149889	newly 3 weeks cumul	16287.	20028.1	{19396., 20800.}	22.9696	0.0894495	newly 5 weeks spec	1653.	2929.56	{2770., 3115.}	77.2269	0.247082	newly 5 weeks cumul	20536.	27126.9	{26465., 28078.}	32.0946	0.120424	newly 55 weeks cumul	22473.	32695.4	{32080., 33781.}	45.4875	0.162199
24	newly 3 weeks spec	2779.	1758.35	{1484., 2003.}	36.7272	0.201848	newly 3 weeks cumul	13850.	11301.	{9478., 12862.}	18.4674	0.0919016	newly 5 weeks spec	702.	375.6	{260., 491.}	46.4957	0.283591	newly 5 weeks cumul	16098.	12517.4	{10616., 14180.}	22.2425	0.111984	newly 59 weeks cumul	16529.	12845.5	{11101., 14546.}	22.2848	0.111942
25	newly 3 weeks spec	3462.	2845.5	{2538., 3135.}	17.844	0.086904	newly 3 weeks cumul	12350.	10977.5	{9835., 11875.}	11.1643	0.0526086	newly 5 weeks spec	2037.	1406.05	{1217., 1623.}	30.9745	0.164254	newly 5 weeks cumul	17226.	14427.9	{13021., 15625.}	16.2434	0.0780925	newly 50 weeks cumul	22743.	17073.5	{15569., 18381.}	24.9285	0.125776
26	newly 3 weeks spec	3048.	2058.45	{1817., 2298.}	32.4656	0.172104	newly 3 weeks cumul	12655.	10013.2	{9092., 10906.}	20.8758	0.102711	newly 5 weeks spec	1843.	706.07	{577., 852.}	61.6891	0.422303	newly 5 weeks cumul	16877.	11945.4	{10933., 13138.}	29.221	0.150975	newly 52 weeks cumul	20285.	12822.5	{11769., 13852.}	36.7885	0.200034
27	newly 3 weeks spec	3519.	2654.78	{2390., 2941.}	24.5587	0.12401	newly 3 weeks cumul	12256.	10501.8	{9544., 11254.}	14.3531	0.0682085	newly 5 weeks spec	1833.	1248.68	{1062., 1415.}	31.8778	0.169383	newly 5 weeks cumul	16564.	13625.9	{12521., 14595.}	17.7379	0.0857074	newly 50 weeks cumul	21086.	15936.9	{14570., 17175.}	24.4197	0.122598
28	newly 3 weeks spec	2682.	2213.58	{1874., 2525.}	17.4966	0.086039	newly 3 weeks cumul	8965.	8282.37	{7124., 9076.}	8.97613	0.0420962	newly 5 weeks spec	1792.	1193.23	{973., 1382.}	33.4135	0.179777	newly 5 weeks cumul	12944.	11132.4	{9777., 12244.}	14.0674	0.0675588	newly 51 weeks cumul	19275.	13690.7	{12311., 15093.}	28.9717	0.149896
29	newly 3 weeks spec	2188.	2683.23	{2299., 3060.}	23.559	0.0902497	newly 3 weeks cumul	8468.	9564.15	{8416., 10762.}	14.3929	0.0575082	newly 5 weeks spec	1093.	1502.79	{1315., 1704.}	38.8847	0.141356	newly 5 weeks cumul	11210.	13117.4	{11842., 14695.}	17.9987	0.0709179	newly 53 weeks cumul	14641.	16348.5	{15000., 17786.}	13.4431	0.054758
30	newly 3 weeks spec	3476.	3221.63	{3000., 3462.}	7.65334	0.0352268	newly 3 weeks cumul	15376.	14588.6	{13690., 15430.}	5.50982	0.0250149	newly 5 weeks spec	1413.	1155.24	{969., 1318.}	18.3822	0.0913935	newly 5 weeks cumul	19115.	17715.9	{16568., 18669.}	7.34434	0.0335981	newly 55 weeks cumul	20923.	19200.4	{17912., 20155.}	8.24676	0.0378251
31	newly 3 weeks spec	2766.	2823.82	{2441., 3181.}	8.34996	0.0357811	newly 3 weeks cumul	10733.	10688.6	{9587., 11629.}	6.60794	0.0290176	newly 5 weeks spec	1803.	1425.21	{1203., 1671.}	21.152	0.106846	newly 5 weeks cumul	14870.	14174.9	{12806., 15424.}	7.07868	0.0324021	newly 52 weeks cumul	18960.	16815.7	{14842., 18495.}	11.59	0.0549702
32	newly 3 weeks spec	2860.	1783.96	{1534., 2063.}	37.6238	0.208067	newly 3 weeks cumul	9853.	7499.77	{6630., 8350.}	23.8834	0.120121	newly 5 weeks spec	1902.	845.55	{688., 1030.}	55.5442	0.358519	newly 5 weeks cumul	14117.	9590.99	{8474., 10800.}	32.0607	0.169718	newly 50 weeks cumul	21462.	11184.5	{9868., 12405.}	47.8869	0.285091
33	newly 3 weeks spec	1762.	3163.38	{2869., 3438.}	79.5335	0.253123	newly 3 weeks cumul	12781.	17049.8	{15082., 18969.}	33.3999	0.123645	newly 5 weeks spec	298.	705.65	{550., 887.}	136.795	0.36597	newly 5 weeks cumul	13856.	19285.7	{17540., 20977.}	39.1869	0.142583	newly 60 weeks cumul	13996.	19837.5	{18283., 21385.}	41.7372	0.150665
34	newly 3 weeks spec	2684.	2969.9	{2736., 3185.}	10.7556	0.0437502	newly 3 weeks cumul	12876.	14000.3	{13068., 14874.}	9.03433	0.0371229	newly 5 weeks spec	799.	1009.62	{877., 1123.}	26.6533	0.100796	newly 5 weeks cumul	15119.	16781.3	{15691., 17738.}	11.0953	0.0452692	newly 56 weeks cumul	15839.	18045.4	{16982., 19088.}	13.93	0.0562162
35	newly 3 weeks spec	685.	831.45	{631., 1022.}	24.9474	0.0944291	newly 3 weeks cumul	10337.	12373.9	{10182., 14240.}	21.7732	0.0844302	newly 5 weeks spec	47.	70.7	{21., 140.}	76.2553	0.266373	newly 5 weeks cumul	10552.	12676.3	{10466., 14576.}	21.8683	0.084748	newly 60 weeks cumul	10552.	12700.7	{10474., 14578.}	22.0497	0.0853734
36	newly 3 weeks spec	2872.	3469.51	{2479., 4013.}	26.0658	0.100626	newly 3 weeks cumul	12863.	14374.6	{11630., 16361.}	15.8077	0.0639155	newly 5 weeks spec	910.	1332.25	{794., 1637.}	50.6077	0.173773	newly 5 weeks cumul	15281.	17919.5	{14242., 20130.}	20.7003	0.0813588	newly 60 weeks cumul	16833.	19656.2	{15233., 21991.}	21.1148	0.0835229
37	newly 3 weeks spec	3362.	1756.72	{1412., 2056.}	47.7478	0.286541	newly 3 weeks cumul	13277.	9427.54	{7825., 10787.}	28.9934	0.15199	newly 5 weeks spec	1507.	529.46	{351., 698.}	64.8666	0.468649	newly 5 weeks cumul	17055.	10931.7	{9038., 12529.}	35.9034	0.196174	newly 53 weeks cumul	19554.	11520.1	{9687., 13129.}	41.0857	0.232647
38	newly 3 weeks spec	2972.	2830.42	{2157., 3259.}	10.7577	0.0510232	newly 3 weeks cumul	16469.	15207.3	{12146., 17520.}	11.1712	0.0536485	newly 5 weeks spec	612.	646.35	{397., 876.}	25.9199	0.117101	newly 5 weeks cumul	18339.	17235.8	{13881., 19313.}	9.64922	0.0457647	newly 60 weeks cumul	18646.	17805.9	{14791., 19855.}	8.90245	0.0417534
39	newly 3 weeks spec	2387.	1831.33	{1554., 2104.}	23.3	0.117805	newly 3 weeks cumul	10499.	9552.54	{8225., 10647.}	10.036	0.0473089	newly 5 weeks spec	937.	583.87	{470., 686.}	37.6873	0.210643	newly 5 weeks cumul	12897.	11180.9	{9798., 12399.}	13.4392	0.0643389	newly 57 weeks cumul	14107.	11885.7	{10554., 13081.}	15.7463	0.0759118
40	newly 3 weeks spec	2220.	1779.85	{1579., 2014.}	19.8329	0.0979457	newly 3 weeks cumul	13427.	11930.5	{10827., 13020.}	11.3052	0.0531792	newly 5 weeks spec	616.	375.76	{286., 457.}	39.	0.222707	newly 5 weeks cumul	15217.	13123.3	{11985., 14182.}	13.7591	0.0652866	newly 59 weeks cumul	15737.	13426.7	{12411., 14502.}	14.6809	0.0698892
41	newly 3 weeks spec	3803.	3616.78	{3439., 3807.}	5.34946	0.0243629	newly 3 weeks cumul	18778.	19090.3	{17991., 20168.}	4.12776	0.0177154	newly 5 weeks spec	894.	940.95	{823., 1099.}	10.5134	0.0447791	newly 5 weeks cumul	21558.	21915.4	{20770., 22913.}	3.6174	0.0155808	newly 60 weeks cumul	22565.	22791.1	{21759., 23773.}	3.1723	0.0137719
42	newly 3 weeks spec	3308.	1152.94	{905., 1406.}	65.1469	0.465466	newly 3 weeks cumul	14867.	9345.51	{7119., 11699.}	37.1392	0.211743	newly 5 weeks spec	1090.	197.21	{86., 322.}	81.9073	0.791126	newly 5 weeks cumul	17792.	10015.9	{7616., 12285.}	43.7058	0.257914	newly 57 weeks cumul	18900.	10149.2	{7644., 12416.}	46.3007	0.277474
43	newly 3 weeks spec	4665.	2860.36	{2244., 3566.}	38.6847	0.219837	newly 3 weeks cumul	19701.	15653.5	{13179., 18334.}	20.5675	0.103277	newly 5 weeks spec	1372.	682.26	{449., 946.}	50.621	0.325409	newly 5 weeks cumul	23754.	17770.	{15036., 20674.}	25.1949	0.129511	newly 60 weeks cumul	25089.	18397.3	{15669., 21412.}	26.7257	0.138492
44	newly 3 weeks spec	3246.	4111.9	{3238., 5169.}	27.1639	0.096182	newly 3 weeks cumul	17555.	19964.4	{17246., 23063.}	14.6142	0.0568383	newly 5 weeks spec	782.	1236.63	{815., 1741.}	59.0985	0.182081	newly 5 weeks cumul	19996.	23516.1	{20046., 27686.}	18.0326	0.0682578	newly 59 weeks cumul	20762.	24818.5	{20962., 29654.}	19.8292	0.0739344
45	newly 3 weeks spec	3565.	2372.02	{2060., 2671.}	33.4637	0.179285	newly 3 weeks cumul	13069.	10549.1	{9172., 11797.}	19.2812	0.0948512	newly 5 weeks spec	1772.	891.92	{732., 1031.}	49.6659	0.302905	newly 5 weeks cumul	17480.	12938.9	{11374., 14281.}	25.9788	0.132264	newly 55 weeks cumul	20564.	14165.2	{12435., 15467.}	31.1165	0.163384
46	newly 3 weeks spec	4749.	2975.36	{2203., 4364.}	37.3797	0.219649	newly 3 weeks cumul	18136.	14393.9	{11764., 17952.}	21.0501	0.107911	newly 5 weeks spec	1998.	953.44	{509., 1627.}	52.2803	0.362894	newly 5 weeks cumul	23379.	17085.5	{13721., 22314.}	26.9574	0.144268	newly 56 weeks cumul	26044.	18191.5	{14519., 24527.}	30.151	0.165186
47	newly 3 weeks spec	3376.	2839.52	{2001., 3478.}	17.5444	0.0935353	newly 3 weeks cumul	16086.	14678.5	{12162., 16451.}	10.166	0.0487983	newly 5 weeks spec	1272.	839.82	{419., 1164.}	34.033	0.218923	newly 5 weeks cumul	19497.	17101.8	{13592., 19655.}	12.9717	0.0645106	newly 56 weeks cumul	20865.	18049.4	{13985., 20943.}	14.02	0.0710628
48	newly 3 weeks spec	3671.	2294.7	{1918., 2507.}	38.7257	0.21582	newly 3 weeks cumul	14471.	10840.7	{9227., 12156.}	25.6608	0.13107	newly 5 weeks spec	1686.	814.31	{611., 937.}	54.4917	0.347875	newly 5 weeks cumul	18539.	13057.4	{11252., 14475.}	30.5912	0.16058	newly 51 weeks cumul	21237.	14151.3	{12534., 15270.}	34.861	0.187709
49	newly 3 weeks spec	2539.	1810.23	{1360., 2208.}	28.8897	0.156245	newly 3 weeks cumul	11374.	10016.6	{6914., 13332.}	20.291	0.104494	newly 5 weeks spec	806.	445.07	{228., 666.}	45.1104	0.290416	newly 5 weeks cumul	13685.	11374.5	{7861., 14483.}	19.862	0.104928	newly 57 weeks cumul	14608.	11796.7	{8560., 14792.}	20.5715	0.108793
50	newly 3 weeks spec	2519.	2010.41	{1667., 2279.}	20.2291	0.100963	newly 3 weeks cumul	12653.	11580.3	{9792., 13146.}	10.7606	0.0509226	newly 5 weeks spec	744.	480.23	{355., 606.}	35.6358	0.19979	newly 5 weeks cumul	14778.	13059.3	{11210., 14774.}	12.3238	0.0591187	newly 58 weeks cumul	15627.	13505.8	{11730., 15223.}	13.7545	0.066368
